# Cardiac Mitochondria Dysfunction in Dyslipidemic Mice

**DOI:** 10.3390/ijms231911488

**Published:** 2022-09-29

**Authors:** Alicja Braczko, Barbara Kutryb-Zajac, Agata Jedrzejewska, Oliwia Krol, Paulina Mierzejewska, Magdalena Zabielska-Kaczorowska, Ewa M. Slominska, Ryszard T. Smolenski

**Affiliations:** 1 Department of Biochemistry, Medical University of Gdansk, Debinki 1 St., 80-211 Gdansk, Poland; 2 Department of Physiology, Medical University of Gdansk, 80-211 Gdansk, Poland

**Keywords:** mitochondria, hyperlipidemia, heart, nucleotides, mice, LDL

## Abstract

Dyslipidemia triggers many severe pathologies, including atherosclerosis and chronic inflammation. Several lines of evidence, including our studies, have suggested direct effects of dyslipidemia on cardiac energy metabolism, but details of these effects are not clear. This study aimed to investigate how mild dyslipidemia affects cardiac mitochondria function and vascular nucleotide metabolism. The analyses were performed in 3- and 6-month-old knock-out mice for low-density lipoprotein receptor (*Ldlr−/−*) and compared to wild-type C57Bl/6J mice (WT). Cardiac isolated mitochondria function was analyzed using Seahorse metabolic flux analyzer. The mechanical function of the heart was measured using echocardiography. The levels of fusion, fission, and mitochondrial biogenesis proteins were determined by ELISA kits, while the cardiac intracellular nucleotide concentration and vascular pattern of nucleotide metabolism ecto-enzymes were analyzed using reverse-phase high-performance liquid chromatography. We revealed the downregulation of mitochondrial complex I, together with a decreased activity of citrate synthase (CS), reduced levels of nuclear respiratory factor 1 and mitochondrial fission 1 protein, as well as lower intracellular adenosine and guanosine triphosphates’ pool in the hearts of 6-month *Ldlr−/−* mice vs. age-matched WT. The analysis of vascular ecto-enzyme pattern revealed decreased rate of extracellular adenosine monophosphate hydrolysis and increased ecto-adenosine deaminase activity (eADA) in 6-month *Ldlr−/−* vs. WT mice. No changes were observed in echocardiography parameters in both age groups of *Ldlr−/−* mice. Younger hyperlipidemic mice revealed no differences in cardiac mitochondria function, CS activity, intracellular nucleotides, mitochondrial biogenesis, and dynamics but exhibited minor changes in vascular eADA activity vs. WT. This study revealed that dysfunction of cardiac mitochondria develops during prolonged mild hyperlipidemia at the time point corresponding to the formation of early vascular alterations.

## 1. Introduction

Dyslipidemia, especially with increased levels of low-density lipoproteins (LDL), is one of the main causes of atherosclerosis and cardiovascular-related mortality [1,2]. Many studies associated with dyslipidemia and atherosclerosis are focused on vascular alterations that induce limitations in the oxygen supply and prompt organ dysfunction [3]. In turn, metabolic changes in the heart and particularly in cardiomyocytes during dyslipidemia are poorly studied. The heart is an organ that has a high energy demand, and therefore, its continuous supply plays a critical role in maintaining its function [4].

Murine knockouts of apolipoprotein E (*Apoe−/−*) and LDL receptor (*Ldlr−/−*), as well as the hybrid knockout for both genes (*Apoe−/− Ldlr−/−*), are well-established experimental models of dyslipidemia that diligently reflect this pathology in humans [5]. It has been demonstrated that during dyslipidemia and atherosclerosis, cardiac function was attenuated as a consequence of diminished oxygen supply [6]. Nevertheless, in our previous study, adult *Apoe−/− Ldlr−/−* mice showed preserved cardiac function with only subtle diastolic changes [7]. Interestingly, *Apoe−/−* mice displayed comparatively benign cardiac impairment, including hypertrophy and altered cardiac output [8]. It should be highlighted that among the available murine gene knockouts, *Ldlr−/−* is the best model to resemble human hypercholesterolemia, increasing cholesterol levels to 200-300 mg/dl [9]. However, there are still relatively few reports about the cardiac function and metabolism in *Ldlr−/−* mice. 

Mitochondria are responsible for energy metabolism in most types of cells [10]. Cardiac cells are characterized by especially high mitochondria abundance that relates to the highest oxygen and energy turnover [11]. Some studies identified that dyslipidemia contributes to mitochondrial dysfunction, but its full characteristics, especially in cardiac muscle, are not sufficient [12,13]. Recently, we highlighted that adult *Apoe*−/−*Ldlr−/−* mice have improved cardiac mitochondria function expressed as enhanced tricarboxylic acid (TCA) cycle and stimulated mitochondrial biogenesis [14]. On the other hand, single knockout *Ldlr−/−* mice displayed an increased production of mitochondrial reactive oxygen species (ROS) in the heart and, in consequence, disturbances of mitochondria membrane permeability [15]. Furthermore, studies on *ApoE−/−* mice revealed that hypercholesterolemia, a major atherosclerosis risk factor, significantly accelerates damage of mtDNA and protein nitration in the heart [16].

In addition to the essential role of intracellular nucleotides as cellular energy sources, precursors for nucleic acid synthesis, and their function as coenzymes, they are also engaged in purinergic signaling pathways [17]. In extracellular space, nucleotides such as ATP, ADP, or UTP are released from the cells under many pathological stimuli and, via the P2 receptors, trigger pro-inflammatory and atherogenic effects [18]. In the cardiovascular system, extracellular nucleotides are deactivated by cell-surface ecto-nucleotidases that occur on different cell types, including cardiomyocytes, endothelium, or blood cells [19]. These include ecto-nucleoside triphosphate diphosphohydrolase-1 (CD39) and the following ecto-5′-nucleotidase (CD73) that are engaged in the hydrolysis of ATP or UTP via ADP (UDP) and AMP (UMP) to adenosine (uridine) [20]. The nucleotide derivative adenosine is a nucleoside that exerts its function by the stimulation of P1 receptors, which mostly abolish the negative effects of nucleotides [21]. Although, adenosine signal transduction may be downregulated by its extracellular conversion to inosine via ecto-adenosine deaminase (eADA) [20]. A few reports demonstrated the alterations in the activities of CD39 and CD73 in atherosclerotic patients [22]. While our previously published data showed the increased activity of vascular eADA during hypercholesterolemia and atherosclerosis [18,23].

This study aimed to investigate the changes in cardiac mitochondria function and nucleotide pools together with cardiac and vascular function in a mouse model of dyslipidemia (*Ldlr−/−* mice). 

## 2. Results

### 2.1. Diminished Complex I Respiration in Isolated Cardiac Mitochondria of Ldlr−/− Mice

**Figure 1:**
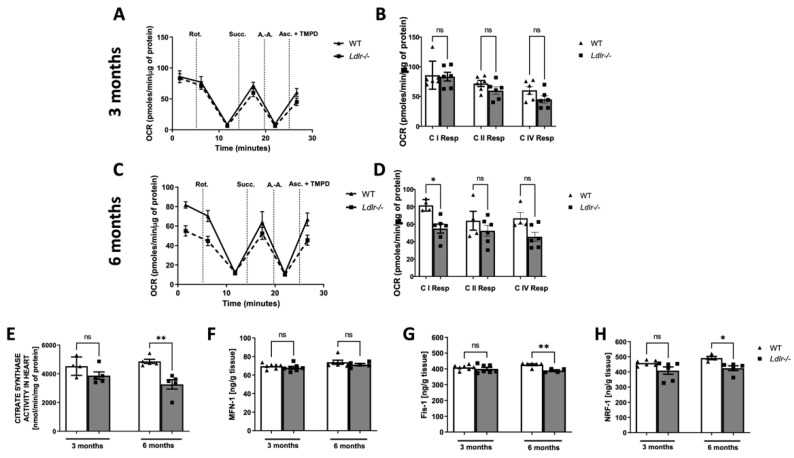
Altered cardiac mitochondria function in a mouse model of dyslipidemia (*Ldlr−/−*). Oxygen consumption rate (OCR) after sequential injection of rotenone (Rot.), succinate (Succ.), antimycin (A.-A.), and ascorbate with tetamethylphenylenediamine (Asc.+TMPD) in isolated cardiac mitochondria of 3-month-old wild-type (WT) and dyslipidemic mice (*Ldlr−/−*) (**A**). The activity of mitochondrial respiratory chain complexes (C) in cardiac mitochondria of 3-month-old WT and *Ldlr−/−* mice (**B**). OCR after sequential injection of Rot., Succ., A.-A., and Asc.+TMPD in isolated cardiac mitochondria of 6-month-old WT and *Ldlr−/−* mice (**C**). The activity of mitochondrial respiratory chain complexes (C) in cardiac mitochondria of 6-month-old WT and *Ldlr−/−* mice (**D**). Complex I and IV respiration (C I Resp, C IV Resp) were calculated as the resulting OCR after subtraction of rotenone or antimycin a driven respiration, respectively. Complex II Respiration (C II Resp) was measured as the succinate-driven OCR. The activity of citrate synthase in the heart of WT and *Ldlr−/−* mice (**E**), and the level of proteins involved in mitochondrial fusion, fission, and mitochondrial biogenesis: mitofusin 1 (MFN-1), mitochondrial fission 1 protein (Fis1), and nuclear respiratory factor 1 (NRF-1) (**F**–**H**), n = 4–7; * *p* < 0.05; ** *p* < 0.01; ns, non-significant.

To clarify the impact of Ldlr knockout on cardiac metabolism, we determined the function of isolated cardiac mitochondria in *Ldlr−/−* and wild-type (WT) mice. Mice were used at 3 and 6 months to determine the mitochondria function at different stages of dyslipidemia. Thus, Oxygen Consumption Rate (OCR) at different levels of the respiratory chain was recorded. We did not observe any differences in I, II, and IV respiration complexes between 3-month-old *Ldlr−/−*and WT mice (Figure 1A,B). Interestingly, cardiac mitochondria isolated from 6-month-old *Ldlr−/−* animals were dysfunctional in comparison to controls (Figure 1C), and complex I respiration was significantly decreased (Figure 1D). In addition, other respiration stages were moderately reduced in cardiac mitochondria of *Ldlr−/−* mice (Figure 1D). Based on the difference in mitochondrial function, we further investigated the activity of citrate synthase (CS), considered a biomarker of mitochondrial abundance. Observed diminished mitochondria function positively correlated with CS activity, which was decreased in 6-month-old *Ldlr−/−* mice (Figure 1E).

Then, to determine the mechanism of cardiac mitochondrial dysfunction, we investigated mitochondrial dynamics evaluating the levels of mitochondrial fission 1 protein (Fis1) and mitofusin 1 (MFN1) that are responsible for fission and fusion processes. The protein level of MFN-1 was similar in all groups (Figure 1F), but cardiac Fis1 concentration was decreased in 6-month-old *Ldlr−/−* mice compared to age-matched control (Figure 1G). Furthermore, we evaluated mitochondrial biogenesis and revealed diminished level of nuclear respiratory factor-1 (NRF-1) in older dyslipidemic mice (Figure 1H). 

### 2.2. Reduced Cardiac Nucleotide Pool in Ldlr−/− Mice 

**Figure 2:**
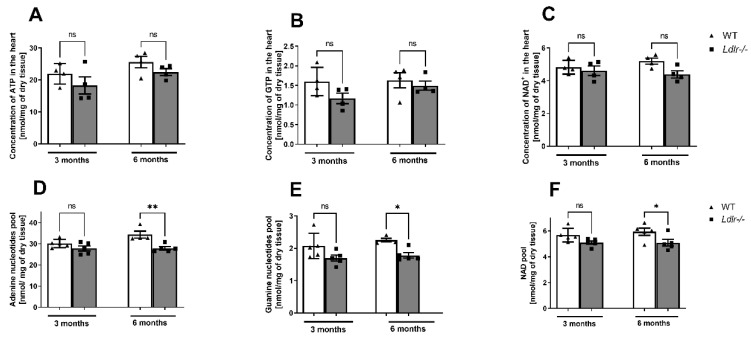
Diminished adenine and guanine nucleotides‘ pool in the hearts of dyslipidemic *Ldlr−/−* mice. The concentration of adenosine triphosphate (ATP) (**A**), guanosine triphosphate (GTP) (**B**), nicotinamide adenine dinucleotide (NAD^+^) (**C**), and adenine (**D**), guanine (**E**), and NAD (**F**) pool in the heart of 3- and 6-month-old dyslipidemic (*Ldlr−/−*) and control (WT) mice, n = 4–5; * *p* < 0.05; ** *p* < 0.01; ns, non-significant.

We further investigated the nucleotide concentrations in the mouse hearts and established the diminished adenine and guanine nucleotides pool in the hearts of 6-month-old *Ldlr−/−* mice in comparison to controls (Figure 2). However, we did not observe differences in the single nucleotide concentrations. Likewise, the nucleotide concentrations in the hearts of the 3-month-old mice remained unmodified.

### 2.3. Alterations in Serum and Liver Lipids in Ldlr−/− Mice

**Figure 3:**
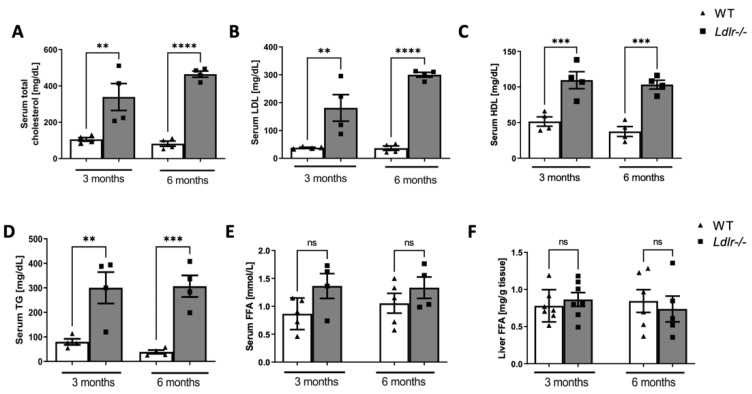
Serum and liver lipids in dyslipidemic *Ldlr−/−* mice. Serum total cholesterol (**A**), LDL cholesterol (**B**) HDL cholesterol (**C**), and triglycerides (**D**) concentration in wild-type (WT) and dyslipidemic (*Ldlr−/−*) mice. Free fatty acids (FFA) in serum (**E**) and liver (**F**) of WT and *Ldlr−/−* mice, n = 4–6; ns, non-significant; ** *p* < 0.01; *** *p* < 0.001; **** *p* < 0.0001.

Determination of serum lipids confirmed the hyperlipidemic phenotype of knockout mice (Figure 3) *Ldlr−/−* mice revealed increased concentrations of serum total, LDL, and HDL cholesterol, together with triglycerides (Figure 3A–D). In turn, dyslipidemic mice did not demonstrate any differences in serum (Figure 3E) and liver (Figure 3F) free fatty acids concentration.

### 2.4. Diminished Branched-Chain Amino Acids (BCAAs), Glutamine, Tyrosine, and Tryptophan in Serum of Ldlr−/− Mice

To elucidate further metabolic alterations in hyperlipidemic mice, we determined the concentration of amino-acid-related compounds in mice serum and revealed the decreased concentration of branched-chain amino acids (BCAA), especially leucine in serum of 6-month-old *Ldlr−/−* mice compared with age-matched control (Table 1). Additionally, 6-month-old *Ldlr−/−* aminals displayed reduced levels of tyrosine, tryptophan, and a tendency to a lower level of phenylalanine. The phenylalanine/tyrosine ratio was slightly increased in the serum of older hyperlipidemic mice compared to WT. Furthermore, the level of glutamine was significantly reduced in the serum of 6-month-old hyperlipidemic mice compared to the control.

### 2.5. Lipid Depositions and Vascular Inflammation in Dyslipidemic Ldlr−/− Mice

**Figure 4:**
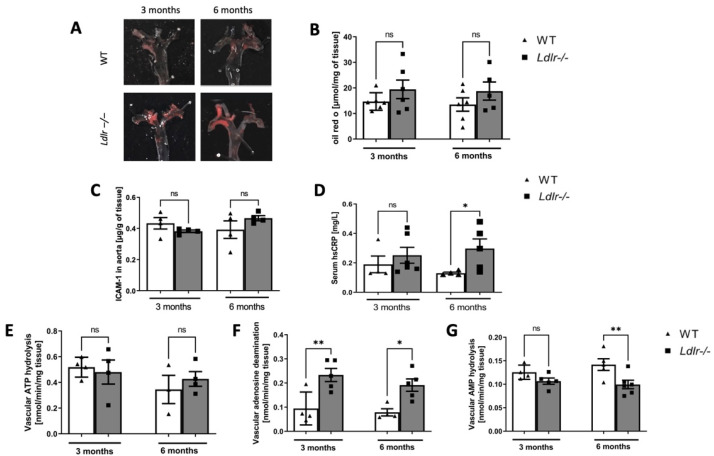
Lipid depositions and vascular inflammation in dyslipidemic *Ldlr−/−* mice. Representative images of aortic arches stained with Oil Red O (**A**) and the concentration of dissolved ORO staining (**B**) from vascular tissue of 3- and 6-month-old wild-type (WT) and dyslipidemic (*Ldlr−/−*) mice. The concentration of intracellular adhesion molecule (ICAM-1) in aortic arch homogenate from WT and *Ldlr−/−* mice (**C**). Serum concentration of high-sensitivity C-reactive protein (hsCRP) in WT and *Ldlr−/−* mice (**D**). Extracellular ATP hydrolysis (**E**), AMP hydrolysis (**F**), and AMP deamination (**G**) in aortic arches isolated from 3- and 6-month-old WT and *Ldlr−/−* mice, n = 4–6; * *p* < 0.05; ** *p* < 0.01; ns, non-significant.

To analyze the severity of vascular abnormalities, we determined the level of lipid depositions in the aortic arch (Figure 4A). Although there were tendencies to a higher concentration of the dissolved Oil red O (ORO) staining from the vascular tissue, there were no significant changes in vascular lipids between *Ldlr−/−* and WT mice (Figure 4B). 

Then, to access the level of vascular and systemic inflammation, we measured the concentrations of vascular intercellular adhesion molecule 1 (ICAM-1), as well as high-sensitivity C-reactive protein (hsCRP) in serum. The concentration of ICAM-1 in the aortic arch homogenates was slightly increased in 6-month-old *Ldlr−/−* mice, while in 3-month-old dyslipidemic mice, it was unchanged (Figure 4C). Similarly, the level of serum hsCRP was increased only in 6-month-old *Ldlr−/−* mice (Figure 4D).

To determine vascular extracellular nucleotide catabolism, the rates of ATP hydrolysis, AMP hydrolysis, and adenosine deamination were determined in the inner surface of *Ldlr−/−* and control mice aortic arch. We did not observe differences in vascular ATP hydrolysis in both age groups of *Ldlr−/−* and controls (Figure 4E), while the rate of vascular AMP hydrolysis was decreased in 6-month-old dyslipidemic mice (Figure 4F). The activity of eADA was three times higher in both 3- and 6-month-old *Ldlr−/−* mice in comparison to WT (Figure 4G). 

### 2.6. Preserved Cardiac Function in Ldlr−/− Mice

**Figure 5:**
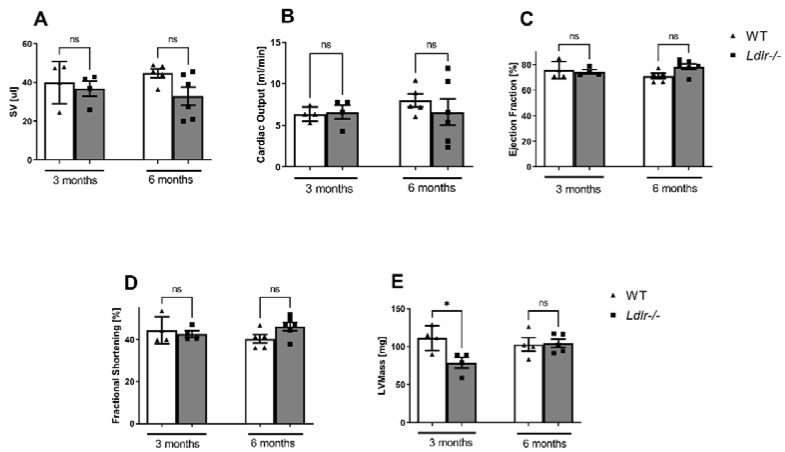
Preserved cardiac function in dyslipidemic *Ldlr−/−* mice. The echocardiographic examination was performed in 3- and 6-month-old dyslipidemic (*Ldlr−/−*) and control (WT) mice. Stroke volume (SV) (**A**), cardiac output (**B**), ejection fraction (**C**), fractional shortening (**D**), and left ventricular mass (LV mass) (**E**) in 3- and 6-month-old animals, n = 4–6; * *p* < 0.05; ns, non-significant.

To characterize the impact of an Ldlr knockout on cardiac mechanical function, echocardiographic measurements were provided. We did not observe the differences between 3-month-old *Ldlr−/−* and WT animals in stroke volume, cardiac output, ejection fraction, and fractional shortening (Figure 5). In turn, left ventricular mass was significantly lower in 3-month-old *Ldlr−/−* compared to WT. In 6-month-old *Ldlr−/−* mice, only a tendency in stroke volume was observed, while the other parameters remained unchanged. 

### 2.7. Increased Red Blood Cells’ Parameters in Ldlr−/− Mice

To greater characterize *Ldlr−/−* mice, we established a blood test in comparison to age-matched control (Table 2). Blood morphology analysis demonstrated a significant increase in red blood cell count in 6-month-old *Ldlr−/−* mice. Moreover, 6-month-old hyperlipidemic mice displayed an increase in hemoglobin, hematocrit, and mean corpuscular volume (MCV). 

## 3. Discussion

This work demonstrated that dyslipidemic *Ldlr−/−* mice develop alterations in the cardiac mitochondria. Mitochondrial dysfunction was accompanied by reduced mitochondrial abundance and biogenesis, disrupted mitochondrial dynamics, and a decrease in cardiac nucleotides’ pool. In addition, systemic and vascular inflammation parameters were upregulated in the dyslipidemic mice model that corresponded with alterations in serum amino acids. However, mechanical heart function remained compensated.

Mouse models are naturally resistant to atherosclerosis due to differences in lipoprotein pattern and cholesterol metabolism [24]. In mice, the lack of cholesteryl ester transfer protein, which is a carrier of triglycerides and cholesteryl esters in the transport between lipoproteins, causes lower levels of circulating LDL cholesterol and makes the HDL the major lipoprotein fraction [25,26]. Hence, to obtain reliable experimental models of hypercholesterolemia and atherosclerosis, genetic modifications have been introduced. It has been demonstrated that C57Bl/6J mice are the only strain that is susceptible to diet-induced hyperlipidemia, and thus it is also commonly used for genetic manipulations that accelerate the formation of atherosclerotic plaques [27]. One of the most popular choices amongst mouse genetic models of atherosclerosis is *Ldlr−/−* mice fed with a Western diet [28]. The receptor for LDL (LDLR) is a glycoprotein occurring on hepatocytes and is engaged in the elimination of circulating LDL cholesterol [29]. In humans, the mutations in the gene encoding LDLR cause familial hypercholesterolemia, with increased serum LDL cholesterol levels and vascular cholesterol depositions [30]. It has been described that *Ldlr−/−* mice on a standard chow diet reflect a human lipoprotein profile, with the cholesterol being mainly confined to the LDL fraction [31]. However, as we proved in this work, 6-month-old *Ldlr−/−* mice on a standard diet spontaneously develop rather mild atherosclerotic lesions, localized in the aortic arch and brachiocephalic artery. Thus, to accelerate the vascular accumulation of foam cells and fatty streaks in *Ldlr−/−* mice, a high-fat diet is essential [32]. This leads to extensive atherosclerotic plaque formation by the further increase in serum LDL cholesterol and triglyceride concentrations [25]. A particularly important role in the research on LDLR deficiency is the analysis of the adipose tissue. It has been found that *Ldlr−/−* mice fed on a high-fat diet developed inflammation of adipose tissue with enhanced IL-6 and TNFα expression [33]. In addition, Wang et al. created a mouse experimental model of generalized lipodystrophy characterized by Seipin/Bscl2 deficiency, one of the genes responsible for congenital generalized lipodystrophy. *Ldlr−/−Seipin−/−* double knockout mice indicated severe hyperlipidemia (total cholesterol about 6000 mg/dl) and spontaneous atherosclerotic plaque formation, which highlights a direct link between LDLR, adipose tissue and plasma cholesterol levels [34]. Interestingly, surgical fat removal in *Ldlr−/−* mice fed on a high-fat diet favored metabolic disorders, but not the development of atherosclerosis [35]. Nonetheless, the main goal of this study was to analyze the energy metabolism of the heart in the state of dyslipidemia that precedes the development of atherosclerosis. In both age groups of the analyzed *Ldlr−/−* mice, we did not observe significant changes in lipids’ accumulation within aortic roots. In addition, vascular and systemic inflammatory parameters were unchanged in younger 3-month-old mice. In turn, the older 6-month-old animals represented increased concentration of vascular ICAM-1 or serum hsCRP. Previously, we have found that adenosine metabolism ecto-enzymes exhibit significant potential as early biomarkers of endothelial activation [18]. In this work, we observed the enhanced activity of vascular eADA in both age groups of *Ldlr−/−* mice, while the rate of extracellular AMP hydrolysis was lower only in 6-month-old dyslipidemic mice. Such an ecto-enzyme pattern decreases the bioavailability of adenosine for the receptor signaling that is critical to maintaining cardiovascular homeostasis [18]. Locally, adenosine interacts with four subtypes of adenosine receptors (ARs) on constituent vascular and cardiac cells: A1, A2A, A2B, and A3ARs. The stimulation of these G-protein-coupled receptors provides many effects, from the modulation of heart rate and coronary flow to cardioprotection, inflammatory regulation, and control of cell growth and tissue remodeling [36]. Cardiovascular adenosine-based therapies including AR agonists are already in place, and trials of new treatments are underway [37]. Importantly, we demonstrated previously that the increased serum cholesterol levels are related to the externalization of membrane eADA in endothelial cells via lipid-dependent exocytosis [23] Thus, it seems that *Ldlr−/−* mice are an adequate model for the study of primary changes induced by long-term moderate hyperlipidemia.

In the heart, ATP is produced by mitochondrial oxidative phosphorylation in 95% while the remaining 5% is covered by glycolysis. Cardiomyocytes use a variety of energy substrates to maintain ATP production including fatty acids, lactate, glucose, ketone, and amino acids [38]. In physiology, 40–60% of ATP production is dependent on fatty acids [39]. Due to the lack of exercise or overeating, lipid abnormalities such as elevated concentrations of cholesterol, triglycerides, or free fatty acids may affect cardiac metabolism leading to cardiomyocyte dysfunction and death [40]. In this study, we observed the reduced mitochondrial complex I respiration in older 6-month-old *Ldlr−/−* mice compared to the controls. On the contrary, our previous study using *Apoe−/− Ldlr−/−* mice at the same age with severe atherosclerotic plaques revealed an unusual improvement in cardiac mitochondria function via stimulated mitochondrial biogenesis [14]. This was in line with our unpublished data where the temporary accelerated cardiac mitochondrial respiration was demonstrated in 6-month-old *Apoe−/− Ldlr−/−* mice. While older double-knockout mice exhibited extensive cardiac mitochondrial dysfunction. This suggests a transient mitochondrial adaptive mechanism in mice with advanced atherosclerosis, which was not observed in mice with moderate hyperlipidemia. It has been shown that the imbalance in the metabolism of energy substrates results in, among others, the depletion of mitochondrial DNA (mtDNA) copy number and accumulation of mtDNA mutations. As a result, such changes lead to cardiac output disturbances [41]. 

Mitochondrial fusion and fission processes play a major role in the maintaining of mitochondria function during environmental or metabolic stresses. Fission leads to the formation of new mitochondria, but also promotes removing of dysfunctional ones and may support apoptosis during cellular stress. Fusion is also essential for rescuing dysfunctional mitochondria by complementation [42]. Disturbances in these mechanisms may affect the functioning of most cells. Fis1 and MFN-1 are proteins involved in fission and fusion processes, respectively. In our study, the level of cardiac Fis1 was decreased in older *Ldlr−/−* mice compared to WT, which suggests a disrupted mitochondrial dynamic after longer periods of dyslipidemia. The disturbance in mitochondrial fission may result in inadequate numbers of mitochondria, which is reflected by decreased activity of citrate synthase, mentioned below. On the other hand, we did not observe differences in MFN-1 cardiac levels, which evidenced no changes in mitochondrial fusion processes in our model. On the contrary, it has been shown that severe hyperlipidemia may lead to mitochondrial dysfunction in the heart via the enhanced fission processes [43]. The experiments on mice and monkeys fed with a high-fat diet resulted in the activation of cardiac dynamin-related protein that is essential for mitochondrial fission, which led to myocardial damage [43]. 

PGC-1α is a coactivator that plays an essential role in a regulatory network of mitochondrial biogenesis and respiratory function [44]. It targets many transcription factors including NRF-1 and NRF-2. PGC-1α expression is regulated by extracellular signals controlling metabolism, and its activity can be modulated by post-translational modification by SIRT1 and AMPK. Here, we demonstrated decreased cardiac level of NRF-1 in older dyslipidemic mice, which indicates disturbed mitochondrial biogenesis [45]. Many reports highlight that impaired myocardial mitochondrial biogenesis leads to reduced efficiency of cardiac energy [46], which is reflected by mitochondrial dysfunction. Conversely, there is a hypothesis that in responding to ATP deficiency, mitochondrial biogenesis could be a compensatory maladaptive mechanism that enhances cardiac dysfunction via overexpression of PGC-1α [47]. Finally, the enhancement in oxidative stress, inefficient mitochondrial biogenesis, and oxidative phosphorylation appear to be abnormalities favoring cardiac dysfunction. 

In this study, we also demonstrated the decreased cardiac activity of CS in 6-month-old *Ldlr−/−* mice, which is a marker of the tissue mitochondrial content [48]. This proved that prolonged hyperlipidemia can lead to the reduction in mitochondrial abundance in the heart. It has been shown that CS activity is essential for the maintenance of metabolic health, and its decreased activity is particularly linked with lipotoxicity [49,50]. In addition to mitochondrial dysfunction, we also observed a diminished adenine nucleotide concentration in the heart of older *Ldlr−/−* mice as compared to controls. The impairment of the respiratory chain in the mitochondria may be associated with disturbances in the oxidative phosphorylation and, consequently, with reduced nucleotide concentrations. Many reports highlight the influence of hyperlipidemia on the disruption of the cardioprotective mechanisms. Csonka et al. found that the cholesterol diet reduces ATP levels in the heart and increases oxidative stress in the cardiac muscle [51]. The decreased ATP pool also has been observed in cardiac tissues received from patients with cardiomyopathy and heart failure [52,53]. 

It has been found that hyperlipidemia affects metabolic dysfunction and stimulates protein utilization, which may disturb the balance of amino acids [54]. The catabolism of branched-chain amino acids (BCAAs), including valine, isoleucine, and leucine, plays a key role in the pathophysiology of metabolic disorders [55]. The catabolic pathway of BCAA starts with reversible transamination into branched-chain alpha-ketoacids by branched-chain amino-transferase and is followed by irreversible oxidative decarboxylation catalyzed by the complex of branched-chain alpha-keto acid dehydrogenase further transform into succinyl-CoA or acetyl-CoA in the TCA cycle [56]. In the present study, serum BCAAs were significantly diminished in 6-month-old *Ldlr−/−* mice compared to controls. Similarly, decreased BCAA concentrations were also found in *Apoe−/−Ldlr−/−* mice [14]. The observed changes in our *Ldlr−/−* mice suggest accelerated utilization of BCAAs and subsequent transport of their metabolites into the TCA cycle. Decreased concentration of valine, one of the essential BCAAs, in serum was also observed in rats on a hypercholesterolemic diet [54]. Moreover, *Ldlr−/−* dams also exhibited significantly decreased concentration of BCAAs, especially valine [57]. Decreased levels of valine in the plasma and aorta as well as its link to cardiovascular risk were also revealed by Martin-Lorenzo et al. [58]. Conversely, Li et al. found an increased concentration of valine in serum of simultaneous LDLR and P-selectin glycoprotein ligand-1 (PSGL-1) deficiency mice [59]. Although, the authors highlighted the impact of PSGL-1 knockout on metabolic regulation of circulating amino acids. Some studies emphasized the accumulation of circulating BCAAs in hyperlipidemia and the development of cardiac dysfunction. Increased levels of BCAAs lead to the disruption of mitochondrial pyruvate utilization by the inhibition of the pyruvate dehydrogenase complex in the heart [56]. Importantly, disrupted BCAA catabolism can intensify mitochondrial dysfunction via the accumulation of toxic BCAA-metabolites [60]. One of the possible explanations for alterations in serum BCAA is a reduced BCAA catabolic flux, observed in obesity [60]. Restoring BCAA catabolism in dysfunctional hearts had therapeutic potential in experimental models [61]. Some reports have indicated that the accumulation of BCAAs in the blood may prelude cardiac dysfunction [62]. However, recent data showed that only an elevated level of circulating isoleucine is linked with an accelerated risk of cardiovascular disease [63]. Thus, the mechanisms underlying differences in serum BCAA levels in *Ldlr−/−* mice require further investigation.

In this study, hyperlipidemic mice displayed diminished concentration of tryptophan and other aromatic amino acids in circulating blood. Metabolism of tryptophan plays an important role in cardiovascular pathologies, particularly via the kynurenine pathway [64]. One of the main metabolites of tryptophan, 3-hydroxyanthranilic, has an anti-inflammatory and anti-atherogenic function [65]. Inhibition of its endogenous degradation contributes to the reduction in atherosclerotic plaque formation, as demonstrated in *Ldlr−/−* mice on a Western diet [66]. 

Interestingly, 6-month-old *Ldlr−/−* mice have displayed increased red blood cell parameters. An accelerated number of erythrocytes was also observed in *Apoe−/−Ldlr−/−* mice with preserved cardiac function [67]. These changes may point to the increased production of erythropoietin (EPO), which plays a cardioprotective role by reducing myocyte apoptosis and the level of pro-inflammatory cytokines [68]. Another mechanism involved in EPO enhancement might be the activation of EPO gene transcription via hypoxia-inducible factors (HIFs) [69]. It has been shown that HIF-1α is engaged in lipid metabolism [70], and its constitutive activation promotes the development of atherosclerosis in *Ldlr−/−* mice [71]. However, our *Ldlr−/−* mice demonstrated preserved cardiac function, which rather suggests the presence of early changes in cardiac metabolism.

## 4. Materials and Methods

### 4.1. Animal Maintenance

Male C57BL/6J (Wild-Type; WT) and low-density lipoprotein receptor knockout (*Ldlr−/−*) mice, both aged three and six months, were used in this study. Water and a standard chow diet were provided ad libitum. All experiments were conducted following the Guide for the Care and Use of the Laboratory Animals published by the European Parliament, Directive 2010/63/EU, and after approval of the Local Ethical Committee for animal experiments in Bydgoszcz (40/2019). *Ldlr−/−* mice (n = 40) were housed in individually ventilated cages with environment control (55 ± 10% humidity, 23 ± 2 °C), with a light/dark 12 h/12 h cycle. Three- and six-month-old mice were used for the experiments.

### 4.2. Cardiac Mitochondria Function 

The isolation of mitochondria from the heart of mice was performed based on a published procedure with some modifications [72]. Three- and six-month-old WT and dyslipidemic mice were anesthetized with isoflurane and then sacrificed by cervical dislocation. The hearts were removed and placed into an ice-cold isolation buffer containing 210 mM mannitol, 70 mM sucrose, 5 mM HEPES, and 1 mM EGTA, and 0.5% (*w*/*v*) fatty acid-free BSA (pH 7.2) was added on the day of analysis. The blood was removed, and subsequently, cardiac tissue was cut into small pieces using a scalpel and scissors. All the following steps of preparation were made on the ice. The tissue was manually homogenized in an isolation buffer enriched with BSA using a glass homogenizer. The obtained homogenate was centrifuged (500× *g*, 4 °C, 10 min) and then fat was carefully discarded, and the remaining supernatant was transferred to a separate tube and centrifuged at 10,000× *g* for 10 min at 4 °C. After centrifugation, the supernatant was discarded and the obtained pellet was resuspended in 1 mL of isolation buffer without BSA to measure the mitochondrial protein. The protein was determined using the Bradford Assay reagent (BioRad) according to the manufacturer’s procedure.

The electron flow assay was performed using Seahorse XFp analyzer. Mitochondrial assay solution (MAS, 1X) was prepared before measurements and it contained 220 mM mannitol, 70 mM sucrose, 10 mM KH_2_PO_4_, 5 mM MgCl_2_, 2 mM HEPES, 1 mM EGTA, and 0.2% (*w*/*v*) fatty acid-free BSA added freshly on the day of assay. MAS was adjusted to pH 7.2 by using 2 M potassium hydroxide (KOH). Substrates for the assay such as succinate, malate, ascorbate, and N,N,N′,N′-Tetramethyl-p-phenylenediamine (TMPD) were diluted in water and adjusted to pH 7.2 with 2 M KOH. Reagents such as Carbonyl cyanide-p-trifluoromethoxyphenylhydrazone (FCCP), rotenone, oligomycin, and antimycin A were diluted in 95% ethanol. All reagents were prepared before analysis and kept frozen at −20 °C, except pyruvate, which was made up freshly on the day of assay. 

Isolated cardiac mitochondria were diluted in cold MAS enriched with 10 mM pyruvate, 2 mM malate, and 4 µM FCCP. Then, 25 µL mitochondrial suspension was placed into Seahorse plate wells (from B to G), and the plate was centrifuged at 2000× *g* for 15 min at 4 °C. The concentration of mitochondrial protein was 6 µg per well. After centrifugation, 180 µL of prewarmed MAS buffer supplemented with pyruvate, malate, and FCCP was added to each well, and the plate was then placed into a non-CO_2_ incubator for 8 min. The Seahorse cartridge was filled with the following reagents: port A, 25 µL of 20 µM Rotenone (2 µM final); port B, 25 µL of 100 mM succinate (10 mM final); port C, 25 µL of 40 µM Antimycin A (4 µM final), port D, 25 µL mix of 100 mM ascorbate and 1 mM TMPD (10 mM and 100 µM final).

### 4.3. Measurement of Citrate Synthase Activity

To measure the activity of citrate synthase, the hearts of dyslipidemic and control mice were weighed and placed in an ice-cold 20 mmol/L Tris hydrochloride buffer (pH 7.8) containing 0.2% Triton X-100 and protease inhibitor cocktail. The tissue-to-buffer ratio was 1:20 (*w*:*v*). The heart was finely cut with scissors and then homogenized. The obtained homogenate was centrifuged at 13,000× *g* for 10 min at 4 °C. The supernatant was collected and used to determine the activity of citrate synthase. The enzyme assay was performed as described before [73]. The buffer used for the assay contained 100 mM Tris-HCl pH 8.1, 0.1 mM 5.5-dithio-bis-2-nitro-benzoic acid (DTNB), 0.5 mM acetyl coenzyme A sodium salt, and 0.5 mM Oxaloacetic acid. Each analysis was performed in duplicate at 37 °C using a Beckman DU 68 spectrophotometer. The absorbance measurements were read at 412 nm.

### 4.4. Cardiac Protein Levels 

Nuclear respiratory factor 1 (NRF-1), mitofusin 1 (MFN-1), and mitochondrial fission 1 protein (Fis1) in the hearts’ homogenates were established by enzyme-linked immunosorbent assay (ELISA) according to the manufacturer’s protocols (Wuhan EIAab Science Co., Wuhan, China). 

### 4.5. The Concentration of Nucleotides in the Heart

The level of cardiac nucleotides was determined by the previously described procedure [74]. Mice were anesthetized with a mixture of ketamine (100 mg/kg), xylazine (10 mg/kg), and 0.9% sodium chloride by intraperitoneal injection. After opening the chest, hearts were immediately frozen in liquid nitrogen and freeze-dried. Subsequently, tissues were homogenized in 0.4 mol/L HClO_4_ using a glass homogenizer. The ratio between heart and homogenization buffer was 1:25 (*w*:*v*). Then, the obtained homogenate was centrifuged at 20,800× *g* at 4 °C for 10 min, and the supernatant was brought to pH 6.0–6.5 using 2 M KOH. The samples were incubated on ice for 15 min and centrifuged at 14,000 rpm (4 °C, 10 min), and the concentration of nucleotides was measured in the supernatants by Reversed-Phase High-Performance Liquid Chromatography (RP-HPLC).

### 4.6. The Concentration of Amino Acids (AAs) in Serum 

The concentration of AAs in serum was measured as described previously [75]. Briefly, 5 μL of the internal standard was added to 25 μL of serum and 70 μL of Acetonitrile. Then, samples were incubated on ice for 20 min and centrifuged at 16,000× *g* (4 °C, 10 min). Subsequently, obtained supernatant was evaporated. The residue was reconstituted in 25 μL of water and analyzed by combined liquid chromatography/mass spectrometry (LC/MS). 

### 4.7. Blood Analysis

After anesthesia, peripheral blood was collected from the inferior vena cava in tubes containing EDTA and clot tubes. Lipids and blood parameters were measured by the ABC Vet analyzer according to the manufacturer’s instructions. High-sensitivity C-reactive protein (hs-CRP) was determined using ERBA autoanalyzer. 

### 4.8. The Concentration of Free Fatty Acids (FFAs) in Serum and Liver Homogenates

To evaluate FFAs in plasma and liver of WT and *Ldlr−/−* mice, the Free Fatty Acid Quantitation Kit (Sigma-Aldrich, MAK044) was used according to the manufacturer’s protocol. 

### 4.9. Quantification of Atherosclerotic Lesions

The atherosclerotic lesions were quantified in *Ldlr−/−* and WT mice aorta by staining with the neutral lipid-targeting lysochrome Oil red O (ORO), solubilizing and measuring the dye maintained by the aortic tissue as described before [76].

### 4.10. The Activity of Ecto-Adenosine Deaminase (eADA), CD73, and CD39 on the Surface of the Aortic Arch

The aortic arch was isolated from the *Ldlr−/−* and WT mice. The vessels were rinsed three times with Hank’s Balanced Salt Solution (HBSS, Sigma Aldrich No H6648) and placed in a 24-well plate containing 1 mL of HBSS. The activity of ecto-adenosine deaminase, CD73, and CD39 was determined as described previously [18]. Briefly, the vessels were pre-incubated for 15 min. Next, adenosine was added to each well to a final concentration of 50 µM. To examine eADA, 50 µL of supernatants were collected in 0, 5, 15, and 30 min. Subsequently, vessels were rinsed three times and pre-incubated in 1 mL of HBSS at 37 °C for 15 min with erythro-9- (2-hydroxy-3-nonyl) adenine (EHNA) (final concentration 5 μM). EHNA was added to inhibit the activity of adenosine deaminase. Subsequently, ATP or AMP was added (final concentration 50 μM), and vessels were incubated at 37 °C for 30 min. An amount of 50 µL of supernatant was collected at 0, 5, 15, and 30 min time points. Obtained samples were immediately analyzed by RP-HPLC.

### 4.11. Echocardiography

An echocardiographic study was determined as described before [77]. Briefly, animals were anesthetized intraperitoneally (i.p) with a mixture of ketamine (100 mg/kg) and xylazine (10 mg/kg). Then, the mice were under deep anesthesia and situated on a heating pad to maintain their body temperature at 37 °C. The echocardiographic examination was accomplished with a high-resolution ultrasound system (Vevo 1100, VisualSonics Inc, Toronto, Canada). The probe (12 MHz) was placed over the anterior chest wall and directed to the ascending aorta in 2D mode. The conducted study allowed us to obtain parameters such as stroke volume (SV), left ventricular mass (LVmass), cardiac output (CO), ejection fraction (EF), and fractional shortening (FS).

### 4.12. Statistical Analysis

Statistical analysis was performed by InStat software (GraphPad, San Diego, CA, USA). The exact value of n was provided for each type of experiments. Error bars indicated the standard error of the mean (SEM). Statistical significance was assumed at *p* ≤ 0.05. Comparisons of mean values between groups were evaluated by two-way analysis of variance (Anova) followed by Holm–Sidak post hoc test.

## 5. Conclusions

The most relevant finding of this study is a decreased function of cardiac mitochondria in *Ldlr−/−* mice with the simultaneous preservation of mechanical heart function. The reduced mitochondrial function correlated with the decreased cardiac mitochondria content, mitochondrial biogenesis, disrupted fission process, and diminished nucleotides’ pool in cardiomyocytes. This work highlights the use of the mouse experimental model of long-term dyslipidemia to study the effects of new therapies on diverse aspects of dyslipidemic pathologies.

## Figures and Tables

**Table 1:** Serum amino acids in *Ldlr−/−* mice. Serum amino acids (AAs) in 3- and 6-month-old dyslipidemic (*Ldlr−/−*) and wild-type (WT) mice, n = 4–7; * *p* < 0.05; ** *p* < 0.01.

Amino Acid [μmol/L]	WT	*Ldlr−/−*	WT	*Ldlr−/−*
Age	3-Month-Old		6-Month-Old	
Alanine	261.30 ± 30.55	376.30 ± 52.80	390.70 ± 101.40	224.80 ± 31.92
Glycine	385.50 ± 95.91	342.70 ± 105.40	467.9 ± 105.20	263.5 ± 57.66
Valine	529.3 ± 29.94	503.8 ± 78.42	558.9 ± 28.03	443.0 ± 28.39 *
Isoleucine	119.10 ± 13.45	101.50 ± 14.26	143.5 ± 14.44	116.60 ± 9.57
Leucine	136.6 ± 11.34	104.7 ± 12.55	153.2 ± 2.46	110.6 ± 6.21 *
BCAAs	776.0 ± 44.41	646.4 ± 81.75	855.6 ± 32.09	670.2 ± 32.83 **
Tryptophan	63.45 ± 6.88	48.53 ± 4.11	74.51 ± 3.23	41.35 ± 3.71 **
Phenylalanine	139.1 ± 14.14	159.4 ± 32.60	159.8 ± 20.50	96.86 ± 7.16 *
Tyrosine	87.80 ± 9.61	66.67 ± 14.54	111.7 ± 17.71	57.59 ± 11.79 *
Phenylalanine/ tyrosine ratio	1.63 ± 0.16	2.00 ± 0.28	1.34 ± 0.16	1.92 ± 0.42
Aspartic acid	25.85 ± 2.72	28.75 ± 5.79	22.41 ± 3.28	26.99 ± 9.12
Glutamic acid	35.49 ± 2.29	48.35 ± 6.69	44.53 ± 4.37	32.86 ± 0.87
Glutamine	771.4 ± 67.8	842.9 ± 72.1	849.3 ± 39.4	530.8 ± 40.2 **
Arginine	155.90 ± 13.27	159.00 ± 9.23	157.20 ± 28.00	150.20 ± 9.36
ADMA	0.55 ± 0.07	0.60 ± 0.09	0.52 ± 0.05	0.53 ± 0.05
SDMA	0.33 ± 0.05	0.41 ± 0.04	0.42 ± 0.05	0.31 ± 1.08
Arginine/ADMA	21.73 ± 0.09	22.50 ± 0.27	21.80 ± 0.27	21.98 ± 0.26
L-NMMA	0.22 ± 0.03	0.23 ± 0.02	0.20 ± 0.04	0.13 ± 0.03
Histidine	84.38 ± 10.65	79.28 ± 6.48	89.18 ± 9.50	69.79 ± 6.61
Lysine	95.79 ± 15.04	82.99 ± 11.35	99.45 ± 9.38	66.20 ± 4.07
Serine	123.4 ± 11.18	162.8 ± 12.04	162.6 ± 29.61	140.2 ± 23.42
Threonine	230.4 ± 25.31	277.8 ± 45.29	273.8 ± 39.06	235.8 ± 17.43
Citrulline	72.07 ± 2.44	75.61 ± 4.60	76.32 ± 6.71	74.75 ± 2.22
Methionine	40.27 ± 3.82	45.77 ± 7.53	52.58 ± 8.26	31.71 ± 5.24
Ornithine	48.11 ± 9.91	47.28 ± 16.12	63.55 ± 6.28	53.28 ± 12.41
Proline	100.60 ± 9.62	145.7 ± 34.83	131.8 ± 20.42	80.78 ± 5.28

**Table 2:** Blood morphology in dyslipidemic Ldlr−/− mice. Body weight and blood morphology parameters in 3-month-old and 6-month-old wild-type (WT) and dyslipidemic mice (Ldlr−/−), n = 10; * *p* < 0.05 vs. 6-month-old WT, ## *p* < 0.01 vs. 3-month-old WT.

Parameter	WT	*Ldlr−/−*	WT	*Ldlr−/−*
Age	3-Month-Old		6-Month-Old	
Body weight [g]	25.00 ± 1.29	19.74 ± 0.92 ##	27.18 ± 1.07	29.24 ± 1.05
Blood morphology				
WBC [G/L]	3.50 ± 0.62	4.80 ± 0.72	4.14 ± 0.72	5.68 ± 0.60
RBC [T/L]	6.94 ± 0.28	6.20 ± 0.33	6.28 ± 0.43	7.49 ± 0.23 *
HGB [mmol/L]	6.84 ± 0.31	6.32 ± 0.34	6.20 ± 0.44	7.42 ± 0.27 *
HCT [%]	33.80 ± 1.06	33.00 ± 1.14	27.83 ± 1.14	32.40 ± 1.16 *
MCHC [mmol/L]	20.99 ± 0.25	21.67 ± 0.36	21.15 ± 0.23	21.48 ± 0.19
MCV [fL]	46.10 ± 0.23	47.90 ± 0.57 ##	44.30 ± 0.37	45.78 ± 0.43 *
MCH [fmol]	0.95 ± 0.01	1.03 ± 0.02 ##	0.92 ± 0.01	0.97 ± 0.01
PLT [G/L]	628 ± 43	459 ± 64	720 ± 62	527 ± 92

## Data Availability

The data presented in this study are available on request from the corresponding author.

## Abbreviations

AA(s)	amino acid(s)
AMP	adenosine monophosphate
*Apoe−/− Ldlr−/−*	apolipoprotein E and low-density lipoprotein receptor double knockout mice
ATP	adenosine triphosphate
BCAA(s)	branched-chain amino acid(s)
CD39	ecto-nucleoside triphosphate diphosphohydrolase-1
CD73	ecto-5′-nucleotidase
CS	citrate synthase
eADA	ecto-adenosine deaminase
EHNA	erythro-9- (2-hydroxy-3-nonyl) adenine
FCCP	carbonyl cyanide-p-trifluoromethoxyphenylhydrazone
FFA(s)	free fatty acid(s)
Fis-1	mitochondrial fission 1 protein
GTP	guanosine triphosphate
HBSS	Hank’s Balanced Salt Solution
HDL	high-density lipoprotein
hsCRP	high-sensitivity C-reactive protein
ICAM-1	intercellular adhesion molecule 1
LDL	low-density lipoprotein
*Ldlr−/−*	low-density lipoprotein receptor knockout mice
MAS	mitochondrial assay solution
MFN-1	mitofusin 1
NRF-1	nuclear respiratory factor 1
OCR	oxygen consumption rate
ORO	oil red O
ROS	reactive oxygen species
TCA cycle	tricarboxylic acid cycle
TG	triglycerides’
TMPD	N,N,N′,N′-Tetramethyl-p-phenylenediamine
WT	wild-type mice; C57BL/6j
